# CO and Soot Oxidation over Ce-Zr-Pr Oxide Catalysts

**DOI:** 10.1186/s11671-016-1494-6

**Published:** 2016-06-02

**Authors:** Tahrizi Andana, Marco Piumetti, Samir Bensaid, Nunzio Russo, Debora Fino, Raffaele Pirone

**Affiliations:** Department of Applied Science and Technology, Politecnico di Torino, Corso Duca degli Abruzzi 24, 10129 Torino, Italy

**Keywords:** Ceria, Mixed oxide catalysts, Zirconia, Praseodymia, Dopants, CO oxidation, Soot oxidation

## Abstract

A set of ceria, ceria-zirconia (Ce 80 at.%, Zr 20 at.%), ceria-praseodymia (Ce 80 at.%, Pr 20 at.%) and ceria-zirconia-praseodymia (Ce 80 at.%, Zr 10 at.% and Pr 10 at.%) catalysts has been prepared by the solution combustion synthesis (SCS). The effects of Zr and Pr as dopants on ceria have been studied in CO and soot oxidation reactions. All the prepared catalysts have been characterized by complementary techniques, including XRD, FESEM, N_2_ physisorption at −196 °C, H_2_-temperature-programmed reduction, and X-ray photoelectron spectroscopy to investigate the relationships between the structure and composition of materials and their catalytic performance. Better results for CO oxidation have been obtained with mixed oxides (performance scale, Ce80Zr10Pr10 > Ce80Zr20 > Ce80Pr20) rather than pure ceria, thus confirming the beneficial role of multicomponent catalysts for this prototypical reaction. Since CO oxidation occurs via a Mars-van Krevelen (MvK)-type mechanism over ceria-based catalysts, it appears that the presence of both Zr and Pr species into the ceria framework improves the oxidation activity, via collective properties, such as electrical conductivity and surface or bulk oxygen anion mobility. On the other hand, this positive effect becomes less prominent in soot oxidation, since the effect of catalyst morphology prevails.

## Background

Ceria has extensively been investigated in many oxidation reactions, thanks to its unique redox properties that allow rapid oxygen intake-uptake [1–3]. Much research has proven the success of ceria in catalyzing soot and CO oxidations [4–10]. In soot oxidation, a ceria-based catalyst requires a good contact with carbon soot; this is normally attained by tuning ceria morphology at a nanoscale level [7, 8, 11–13]. Due to the complexity of solid-gas interaction in soot oxidation reaction, the ability of ceria-based catalysts to initiate active oxygen species is strictly necessary. In CO oxidation, on the other hand, the oxygen storage capacity (OSC), which is high in ceria, plays the crucial role since it has been well understood that CO oxidation over ceria conforms to the Mars-van Krevelen (MvK)-type mechanism [3–5, 14]. In this mechanism, oxygen vacancies are formed during the reaction with CO, followed by oxygen refilling from the bulk phase.

Deficiency of oxygen as the prerequisite of enhanced redox properties is created by introducing metallic dopants [3, 15, 16]. The insertion of dopants perturbs the crystal structure of the catalyst, creating defects that may take form as vacancies or interstitial defects. In general, this may result in more cerium anions in a reduced state (Ce^3+^), improving the OSC of the catalyst. However, different effects might arise depending on the type of the metal. Among the metallic elements frequently investigated as dopants for ceria, zirconium and praseodymium have become of particular interest [6, 11, 15]. Previous experiments with soot oxidation over ceria-zirconia catalysts have shown that the catalytic activity over ceria-zirconia significantly improves [15]. It has also been found that higher dopant content gives better thermal stability (less reducible). However, the cerium redox sites decrease as the dopant concentration increases. Conversely, different phenomena have been observed for praseodymium as dopant for ceria. Our previous work with well-defined nanostructured catalysts has demonstrated that inserting more praseodymium into a ceria lattice framework results in more cerium redox sites, thus increasing the oxygen vacancies. This eventually impacts the catalytic activity, which is the highest for equimolar ceria-praseodymia [11].

In this article, we analyze the effect of zirconium and praseodymium as dopants for ceria-based catalysts. We introduce herein two bi-metallic oxides (ceria-zirconia and ceria-praseodymia) and one tri-metallic oxide (ceria-zirconia-praseodymia) as our testing materials to observe possible synergistic effects among the active species. Therefore, we tested a set of ceria, ceria-zirconia (Ce 80 at.%, Zr 20 at.%), ceria-praseodymia (Ce 80 at.%, Pr 20 at.%), and ceria-zirconia-praseodymia (Ce 80 at.%, Zr 10 at.% and Pr 10 at.%) catalysts for the CO and soot oxidation reactions. Finally, complementary physico-chemical characterizations are provided to accompany the understanding of the catalysts’ properties.

## Methods

### Preparation of Samples

Pure ceria (denoted as Ce100), ceria-zirconia (denoted as Ce80Zr20; Zr atomic percentage = 20 %), ceria-praseodymia (denoted as Ce80Pr20; Pr atomic percentage = 20 %), and ceria-zirconia-praseodymia (denoted as Ce80Zr10Pr10; Pr, Zr atomic percentage = 10 %) were prepared via solution combustion synthesis (SCS) [17]. Therefore, in all ceria-doped catalysts, the total atomic amount of dopants is equal to 20 %. Ce(NO_3_)_3_·6H_2_O, ZrO(NO_3_)_2_·xH_2_O and Pr(NO_3_)_3_·6H_2_O were used as the precursors, all from Sigma-Aldrich. In a typical preparation of the tri-metallic oxide (Ce80Zr10Pr10), 1.5 g of cerium precursor, 0.1 g of zirconium precursor, and 0.2 g of praseodymium precursor were dissolved in 60 mL of water. 0.8 g of urea was then added into the stirring solution. The final clear solution was then transferred into a ceramic crucible which was successively heated in a furnace to 650 °C.

### Characterizations of Samples

Powder X-ray diffractograms of the samples were recorded in an X’Pert Philips PW3040 diffractometer using Cu Kα radiation, 2*θ* range of 20°–70° (angle step size at 0.02°) and a time per step of 0.2 s. The International Centre of Diffraction Data (ICDD) was used as the reference for peak identification. Scherrer’s equation, *D* = 0.9*λ*/*b*cos*θ,* was applied to predict the crystallite size of the sample where *λ* is the wavelength of the Cu K*α* radiation, *b* is the full width at half maximum in radians, 0.9 is the shape factor for spheres, and *θ* is the diffraction peak angle.

N_2_ physisorption of the samples at −196 °C was performed by a Micrometrics ASAP 2020 instrument to determine the specific surface areas and total pore volumes. Removal of water and contaminants from the samples was done prior to analyses by heating the samples at 200 °C for 2 h. The Brunauer-Emmett-Teller (BET) method was applied to calculate the specific surface area of the sample (*S*_BET_).

Field emission scanning electron microscope (FESEM Zeiss MERLIN, Gemini-II column) was used to analyze the morphology of the samples.

H_2_-temperature-programmed reduction (TPR) was performed to analyze the reducibility of the samples. Sample pretreatment was conducted prior to analyses by treating 50 mg of catalyst under air (40 mL min^−1^) at 150 °C for 1 h, followed by cooling with Ar to room temperature. The H_2_-TPR analysis was executed by increasing gradually the sample temperature to 800 °C with the rate of 5 °C min^−1^ under Ar (4.95 %-mol H_2_ in Ar). The instrument was equipped with thermal conductivity detector (TCD) to recognize the H_2_ signal.

X-ray photoelectron spectroscopy measurements were collected on XPS PHI 5000 VersaProbe apparatus using a band-pass energy of 187.85 eV, a 45° takeoff angle, and a 100.0-μm-diameter X-ray spot size. Curve fits were obtained by Multipack 9.0 software.

### Catalytic Tests

CO oxidation tests were carried out in a fixed-bed Quartz reactor (4-mm inner diameter, U-tube) heated by a vertical tubular furnace. In a typical run, 0.1 g of powdered sample was inserted in the reactor. The temperature of the reactor bed was detected by a K-type thermocouple, placed as close as possible to the bed. The test started by continuously flowing 50 mL min^−1^ gas containing 1000 ppm of CO and 50 %-vol air in N_2_ into the reactor. Meanwhile, the furnace heated up at a rate of 5 °C min^−1^ until complete CO conversion was reached. Non-dispersive infrared analyzers were used to record CO_x_ concentrations at the reactor outlet.

Soot oxidation tests were conducted in the same fixed-bed Quartz reactor heated by a vertical tubular furnace. In a typical run, a reactor bed contained 5 mg of soot (Printex-U), 45 mg of powdered sample, and 150 mg of silica. The bed was prepared by ball-milling the solid mixture at 250 rpm for 10 min to get a “tight” contact between the soot and the catalyst. The test started by flowing 100 mL min^−1^ gas comprising of 50 %-vol of air and 50 %-vol of N_2_ to the reactor. The reaction temperature increased from 100 to 700 °C with a 5 °C min^−1^ heating rate. Non-dispersive infrared analyzers were used to record CO_x_ concentrations at the reactor outlet.

## Results and Discussion

### Physical Properties of the Samples

**Fig. 1:**
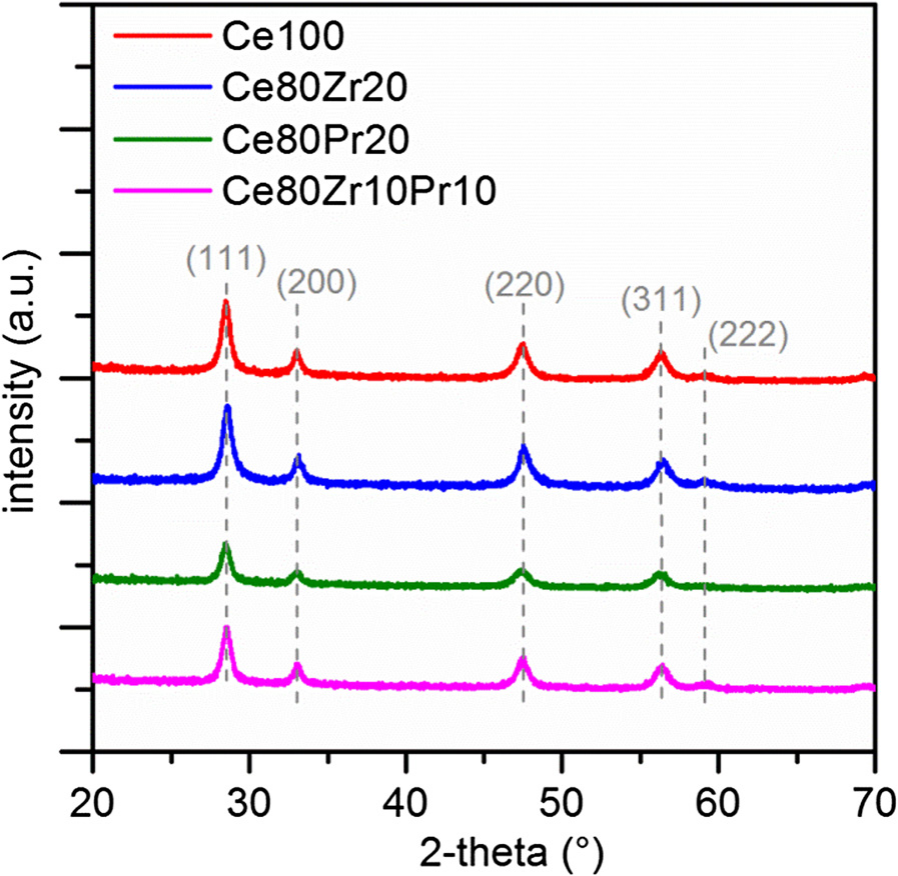
X-ray diffractograms of all prepared samples

Figure 1 shows the X-ray diffractograms of the samples. Analyses with X-ray diffraction prove that all prepared samples refer to a cubic fluorite structure of CeO_2_, indicated typically by (111), (200), (220), (311), and (222) peaks [18, 19]. No additional peaks due to either zirconia (ZrO_2_) or praseodymia (PrO_2_) phases were found in the diffractograms, thus confirming the presence of a single phase for these catalysts.

Simple prediction with Scherrer’s equation using (111) peak as the reference demonstrates the decrease in crystallite size due to the addition of dopants, with the tri-metallic oxide sample having the lowest size (see Table 1). Lattice parameters are modified when dopants are introduced. Zirconia-containing samples experience lattice shrinkage since Zr ionic radii are far lower than those of Ce (ionic radii of Zr^4+^ and Ce^4+^/Ce^3+^ are 0.72 Å and 0.87/1.01 Å) [20]. On the other hand, lattice expansion is found in the ceria-praseodymia sample (Ce80Pr20) despite the similar ionic radii of Ce and Pr (ionic radii of Pr^4+^/Pr^3+^ are 0.85/0.99 Å) [20]. This implies that more 3+ metal ions exist in the sample when Pr is introduced; similar findings have been observed in our previous work [11].

**Table 1:** Physical properties of the samples derived from N_2_ physisorption and X-ray diffraction

Sample	*S*_BET_ (m^2^ g^−1^)	*V*_p_ (cm^3^ g^−1^)	Crystal size (nm)^a^	Lattice parameter (nm)^b^
Ce100	15	0.02	80	5.41
Ce80Zr20	23	0.04	23	5.40
Ce80Pr20	16	0.03	29	5.42
Ce80Zr10Pr10	38	0.06	13	5.41

^a^Average value estimated via Scherrer’s equation

^b^Derived from Rietveld refinement

The BET specific surface areas of the samples derived from N_2_ physisorption can be seen on Table 1. The addition of dopants generally increases the surface area; however, the increment depends much on the inserted metals. Introducing zirconia into ceria structure enlarges the surface area, while embedding only praseodymium into ceria does not modify significantly the overall structure. Again, this might be because both cerium and praseodymium ions are nearly identical in size. The trend for surface area increases as follows: Ce100 < Ce80Pr20 < Ce80Zr20 < Ce80Zr10Pr10.

**Fig. 2:**
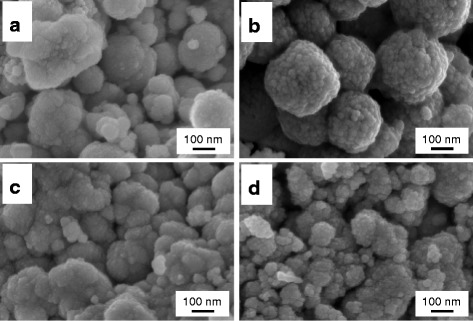
FESEM images of the **a** Ce100, **b** Ce80Zr20, **c** Ce80Pr20, and **d** Ce80Zr10Pr10 samples

Figure 2 shows the morphology of the samples observed through FESEM. Synthesis by solution combustion technique allows the formation of a spongy framework (three-dimensional structures) comprising of small nanoscale agglomerates, thanks to gaseous products rapidly emitted during the reaction. The effect of dopants seems nearly negligible on the overall structure of the samples.

### Reducibility and Redox Properties of the Samples

**Fig. 3:**
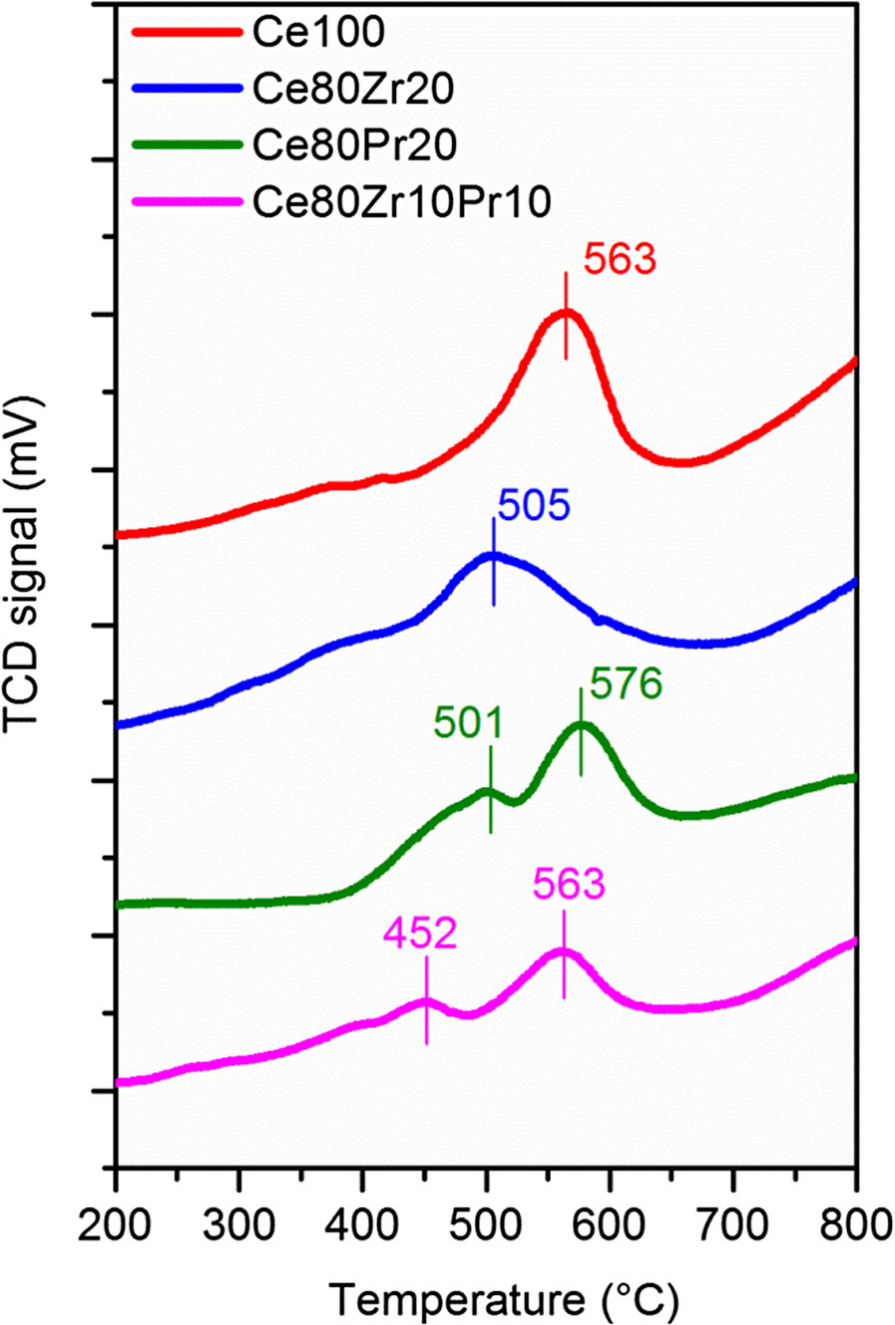
H_2_-TPR profiles of the prepared samples

Figure 3 shows the H_2_-TPR curves for all samples. Reduction profiles of all samples accord generally with the profile of high-surface ceria; the initial reduction occurs at a low temperature (400–600 °C) and the final reduction occurs at a high temperature (>800 °C) [7, 11]. The initial reduction infers the role of surface oxygen, which is weakly attached to the surface of the catalyst. The final reduction is eventually triggered by the slow release of lattice oxygen at an extremely high temperature. The Ce80Zr20 sample has a lower initial reduction peak temperature (505 °C) than the pure ceria sample (563 °C). The Ce80Pr20 sample, on the other hand, exhibits two peaks during the initial reduction: the small one at 501 °C which appears incomplete and then the large one which appears at 576 °C. Although the main peak temperature of ceria-praseodymia sample is higher than that of pure ceria, the reduction commences earlier at a much lower temperature. It is likely that doping with praseodymium favors the weakening of Ce–O bonds, as observed in our previous research [11]. The tri-metallic oxide sample (Ce80Zr10Pr10) is ultimately the most reducible catalyst in the series, having two reduction peaks due to the effect of praseodymium and lower peak temperatures compared to ceria-praseodymia, thanks to the presence of zirconium.

**Fig. 4:**
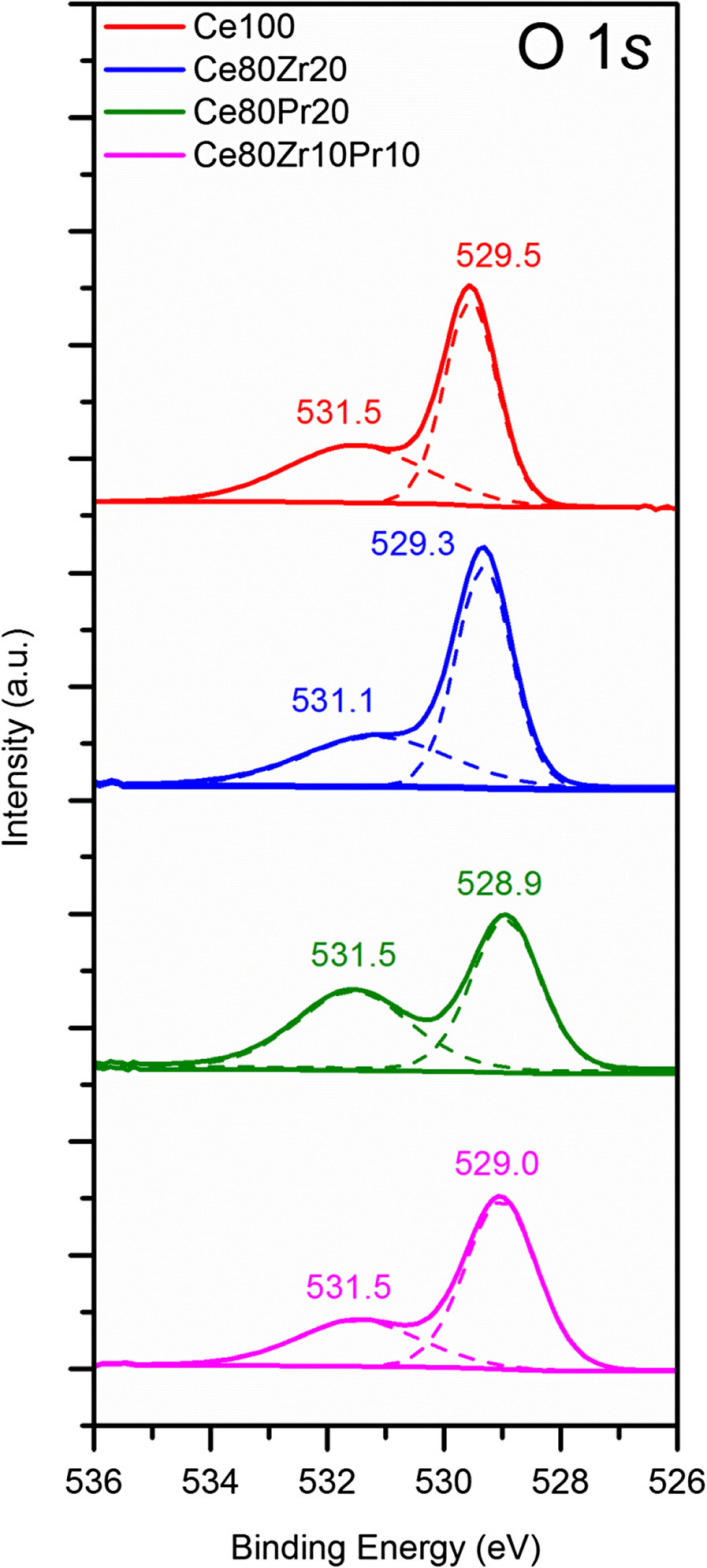
XPS spectra of the samples in the O (1s) core-level regions

**Fig. 5:**
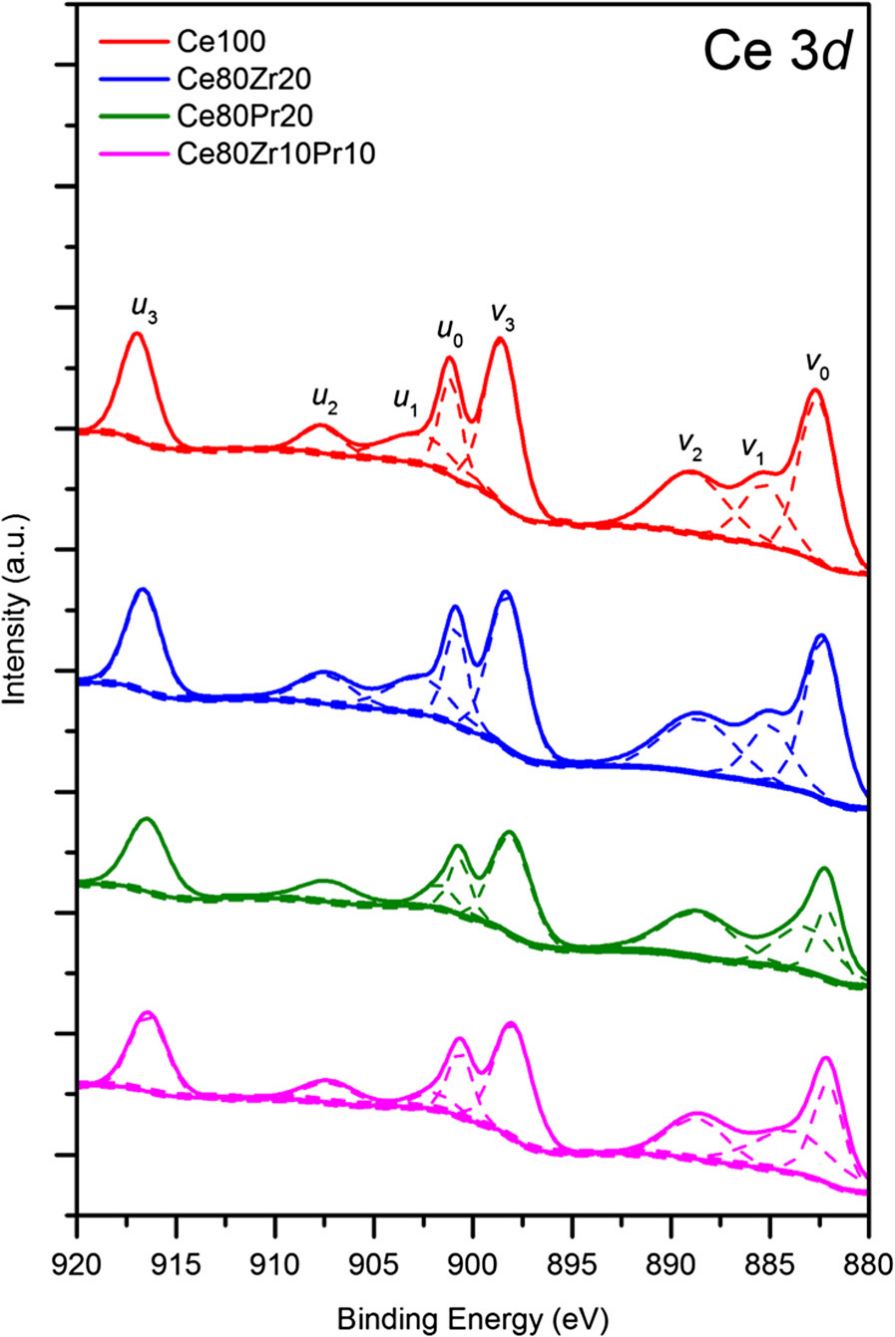
XPS spectra of the samples in the Ce (3*d*) core-level regions

**Fig. 6:**
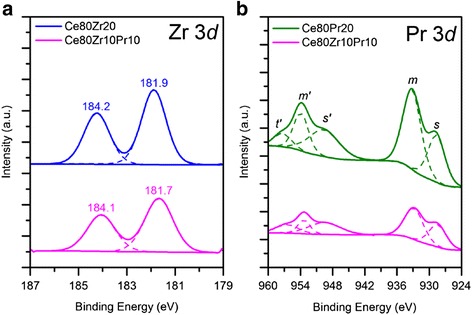
XPS spectra of the samples in the Zr (3*d*) (**a**) and Pr (3*d*) (**b**) core-level regions

Figures 4, 5, and 6 show the XPS spectra in the O (1*s*), Ce, Zr, and Pr (3*d*) BE regions. The O 1*s* spectra (Fig. 4) display the peaks assigned to either chemisorbed oxygen (O_α_ species) or lattice oxygen (O_β_ species). As a whole, the peak at 528.9–529.5 eV corresponds to O_β_ (i.e. O^2−^), whereas the signals at 531.1–531.5 eV are usually due to surface oxygens (e.g. O_2_^2−^, O^−^, OH^−^, CO_3_^2−^) [7, 11, 15]. As reported in Table 2, different O_α_/O_β_ ratios appear for the prepared samples. Interestingly, the Ce80Zr10Pr10 sample exhibits the lowest O_α_/O_β_ ratio, although it has the highest surface area. This suggests that oxidation reactions over the Ce80Zr10Pr10 catalyst might be mediated mainly by the lattice oxygens [6].

**Table 2:** Results of curve fittings on O 1*s* XP spectra of all samples

Sample	O_α_		O_β_		O_α_/O_β_
	BE (eV)	%-atom	BE (eV)	%-atom	
Ce100	531.5	42.3	529.5	57.7	0.73
Ce80Zr20	531.2	37.2	529.3	62.8	0.59
Ce80Pr20	531.6	45.4	528.9	54.6	0.83
Ce80Zr10Pr10	531.5	31.7	529.0	68.3	0.46

​ *BE* binding energy

The dissociative adsorption of molecular oxygen requires steps of electron transfers from the solid catalyst into the anti-bonding *π*-orbital of the molecule until cleavage of the O–O bond is reached [21–23]:

$$ {\mathrm{O}}_{2\mathrm{ads}}\left(+{e}^{-}\right)\ \leftrightarrow\ {\mathrm{O}}_2{{}^{\hbox{-}}}_{\mathrm{ads}}\left(+{e}^{-}\right)\ \leftrightarrow\ {\mathrm{O}}_2{{}^{2\hbox{-}}}_{\mathrm{ads}}\leftrightarrow\ 2{{\mathrm{O}}^{\hbox{-}}}_{\mathrm{ads}}\left(+2{e}^{-}\right)\ \leftrightarrow\ 2{\mathrm{O}}^{2\hbox{-} } $$

This electronic property is beneficial in oxidation catalysis, since the requirements for the MvK mechanism to take place comprise a good mobility of both electrons and lattice oxygen anions within the solid catalyst. Previous research have demonstrated that introducing dopants into the ceria framework facilitates the activation of adsorbed oxygen to form superoxo (O_2_^−^) and peroxo species (O_2_^2−^) [24, 25].

Table 3 reports the relative abundance of the Ce^3+^ and Ce^4+^ species for each sample which is estimated considering the deconvolution results of Ce 3*d* (Fig. 5). As summarized in Table 4, the relative amount of Ce^3+^ species ranges from 16 to 24 at.%. The highest Ce^3+^ content is reached for the Ce80Pr20 sample, thus suggesting the role of Pr^4+^ ions in maintaining a lower oxidation state of Ce species. On the other hand, Zr^4+^ species have an opposite effect on the oxidation state of Ce species [15]. As a result, an intermediate value of the Ce^3+^ abundance can be found for the Ce80Zr10Pr10 sample (namely, 21 at.%). Figure 6a shows the Zr (3*d*) spectra for the Ce80Zr20 and Ce80Zr10Pr10 samples. The peaks at 181.9–182.3 eV correspond to the Zr 3*d*_5/2_ states, whereas the signals at 184.4 eV reflect the Zr 3*d*_3/2_ levels. As a whole, no signal is due to the ZrO_2_ phase (expected at ca. 182.9 eV) [15, 26], further confirming its absence in these mixed oxides (no-extra framework ZrO_2_), in agreement with XRD results. This means that a good incorporation of the Zr species into the ceria framework has been obtained for both samples. The different peak intensities for the two samples is essentially due to the dissimilar Zr loading into the materials (namely, 10 or 20 at.% for the Ce80Zr20 and Ce80Zr10Pr10 samples, respectively). The ratios between the areas of the 3*d*_5/2_ and 3*d*_3/2_ peaks are slightly different for the two samples (see Table 5), reflecting a difference in terms of electronic state of Zr species. Figure 6b shows finally the Pr 3*d* core-level XPS spectra of the Ce80Pr20 and Ce80Zr10Pr10 samples. The peaks in the spectra reflect two different states: (i) Pr 3*d*_5/2_ at lower binding energies (927–933 eV), in which two peaks, *m* and *s*, were designated, and (ii) Pr 3*d*_3/2_ at higher binding energies (952–965 eV), in which four peaks appear (namely, *m’*, *s’*, *t’*, and *f’*). The denotation *m*,*m’* and *s,s’* refers to the “main” and “satellite” peaks, respectively, while *t’* refers to extra-structure, existing only in 3*d*_3/2_ form (Table 6) [27]. On the other hand, the peak *f’*, originated uniquely from the PrO_2_ phase as a marker of Pr^4+^ oxidation state [28, 29], does not occur. This means that in the ceria framework, both Pr^3+^ and Pr^4+^ oxidation states exist. However, the relative abundance of these two species remains a difficult task. In fact, the primary peaks (*m,m’, s,s’, t’*) appear equally in Pr_2_O_3_ and PrO_2_, and hence the ascription of peaks to a definite oxidation state is rather impossible. Nevertheless, a different distribution of the energetic states *m,m’, s,s’,* and *t’* occurs for the two mixed oxides.

**Table 3:** Results of curve fittings on Ce 3*d* XP spectra of all samples

Ce 3*d*_5/2_								
Sample	*v*_0_ (Ce^4+^)		*v*_1_ (Ce^3+^)		*v*_2_ (Ce^4+^)		*v*_3_ (Ce^4+^)	
	BE (eV)	%-atom	BE (eV)	%-atom	BE (eV)	%-atom	BE (eV)	%-atom
Ce100	882.6	22.6	885.3	10.1	889.0	15.0	898.5	20.2
Ce80Zr20	882.3	21.1	885.0	8.94	888.6	16.0	898.3	20.4
Ce80Pr20	882.1	10.6	883.1	17.1	888.6	17.7	898.1	22.7
Ce80Zr10Pr10	882.1	14.7	883.8	17.0	888.7	12.9	898.0	22.2
Ce 3*d*_3/2_								
Sample	*u*_0_ (Ce^4+^)		*u*_1_ (Ce^3+^)		*u*_2_ (Ce^4+^)		*u*_3_ (Ce^4+^)	
	BE (eV)	%-atom	BE (eV)	%-atom	BE (eV)	%-atom	BE (eV)	%-atom
Ce100	901.1	7.73	902.7	7.39	907.7	3.87	916.9	13.1
Ce80Zr20	900.8	7.88	902.7	7.54	907.4	5.66	916.6	12.5
Ce80Pr20	900.7	6.50	901.7	4.01	907.3	5.63	916.4	15.7
Ce80Zr10Pr10	900.6	8.33	901.9	4.02	907.2	5.63	916.4	15.2

**Table 4:** Concentrations of Ce^3+^ (%) estimated from Ce 3*d* XP spectra deconvolution

Sample	Ce^3+^ concentration (%)
Ce100	17.5
Ce80Zr20	16.5
Ce80Pr20	23.6
Ce80Zr10Pr10	21.0

**Table 5:** Results of curve fittings on Zr 3*d* XP spectra of all samples

Sample	Zr^4+^				Ratio
	BE (eV)	%-atom	BE (eV)	%-atom	3*d*_5/2_/3*d*_3/2_
Ce100	–	–	–	–	
Ce80Zr20	181.9	58.4	184.2	41.6	1.40
Ce80Pr20	–	–	–	–	
Ce80Zr10Pr10	181.7	59.8	184.1	40.2	1.48

**Table 6:** Results of curve fittings on Pr 3*d* XP spectra of all samples

Pr 3*d*_5/2_						
Sample	*m* (Pr^3+^/Pr^4+^)			*s* (Pr^3+^/Pr^4+^)		
	BE (eV)		%-atom	BE (eV)		%-atom
Ce100	–		–	–		–
Ce80Zr20	–		–	–		–
Ce80Pr20	933.2		37.3	928.6		20.7
Ce80Zr10Pr10	932.8		34.0	928.4		22.8
Pr 3*d*_3/2_						
Sample	*m’*		*s’*		*t’*	
	BE (eV)	%-atom	BE (eV)	%-atom	BE (eV)	%-atom
Ce100	–	–	–	–	–	–
Ce80Zr20	–	–	–	–	–	–
Ce80Pr20	953.9	14.6	949.6	21.4	957.4	5.97
Ce80Zr10Pr10	953.4	9.82	949.5	22.2	956.5	11.2

### Catalytic Tests

**Fig. 7:**
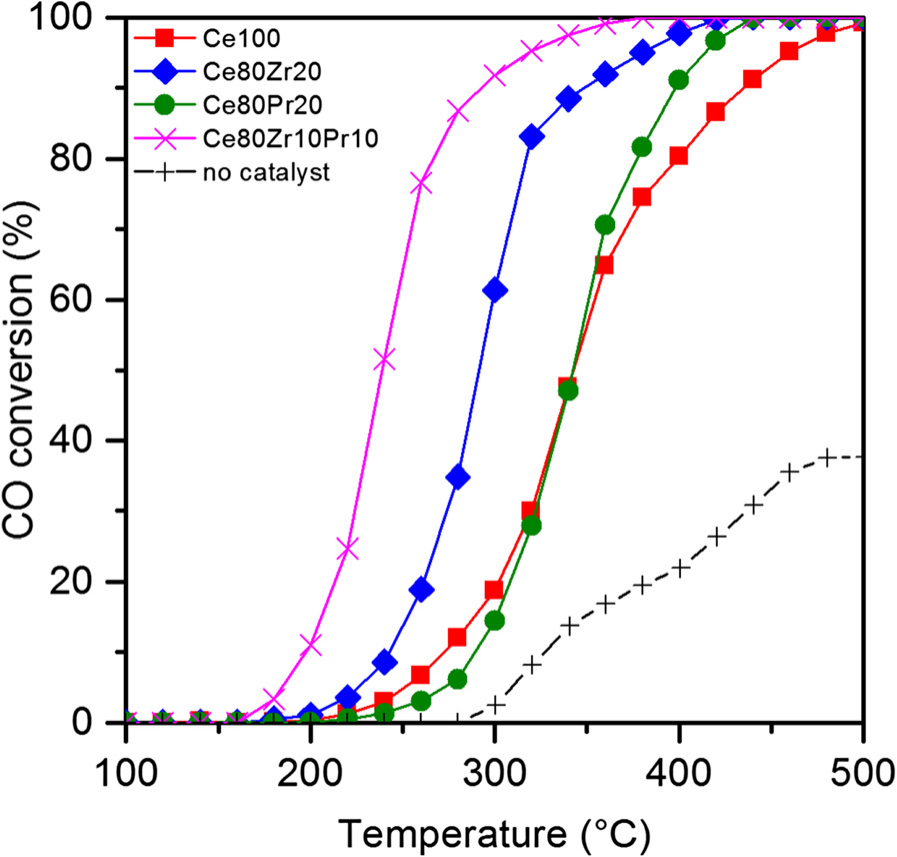
CO conversion to CO_2_ profiles as a function of temperature for the prepared catalysts

Figure 7 summarizes the results of CO oxidation catalytic tests over four samples. The curves indicate the CO conversion to CO_2_ as a function of temperature. In general, our catalysts improve greatly the CO conversion, as only about 40 % of the initial CO converts naturally to CO_2_ at 500 °C. It is evident that the Ce80Zr10Pr10 sample outperforms the other oxides. The tri-metallic oxide sample is able to convert half of the CO reactant at the lowest temperature (*T*_50%_ = 249 °C). This high activity likely draws contributions from the high reducibility, better redox properties, and the average particle size of the catalyst (*vide supra*). Since the MvK-type mechanism governs the reaction of CO oxidation over ceria-based catalysts, the reducibility and redox behavior of the solid catalysts plays a pivotal role in obtaining a high activity. In fact, the more reducible the catalyst, the easier the release of surface oxygen species accessible to CO molecule; moreover, a high electrical conductivity and oxygen anion mobility are beneficial factors for the oxidative mechanism [30, 31]. On the other hand, the geometric factor seems to play a role on the catalytic performances: smaller particle size helps to improve the catalytic activity by providing more accessible active sites to CO molecules [32].

It appears that the activity trend for CO oxidation follows this order: Ce100 < Ce80Pr20 < Ce80Zr20 < Ce80Zr10Pr10 (see Table 7 for complete data). This means that the Ce80Pr20 sample is less active than Ce80Zr20 despite having better reducibility. However, a similar trend has also been encountered in the BET surface areas. This implies the dependence of CO oxidation reaction on surface area. The MvK-type mechanism suggests that the reaction between CO and oxygen specie takes place on the catalyst surface. A higher surface area grants more opportunity for reactions to occur. This reactivity order reflects fairly well the abundance of O_β_-species which is maximum for the Ce80Zr10Pr10 sample, as revealed by XPS data. Thus, a collective (electronic) behavior of the active (redox) sites for the tri-metallic oxide seems to have a beneficial effect on the catalytic reactivity.

**Table 7:** Results from CO oxidation over the prepared catalysts

Sample	*T*_10%_ (°C)	*T*_50%_ (°C)	*T*_90%_ (°C)
No catalyst	327	–	–
Ce100	269	343	436
Ce80Zr20	243	292	352
Ce80Pr20	290	343	399
Ce80Zr10Pr10	197	239	295

**Fig. 8:**
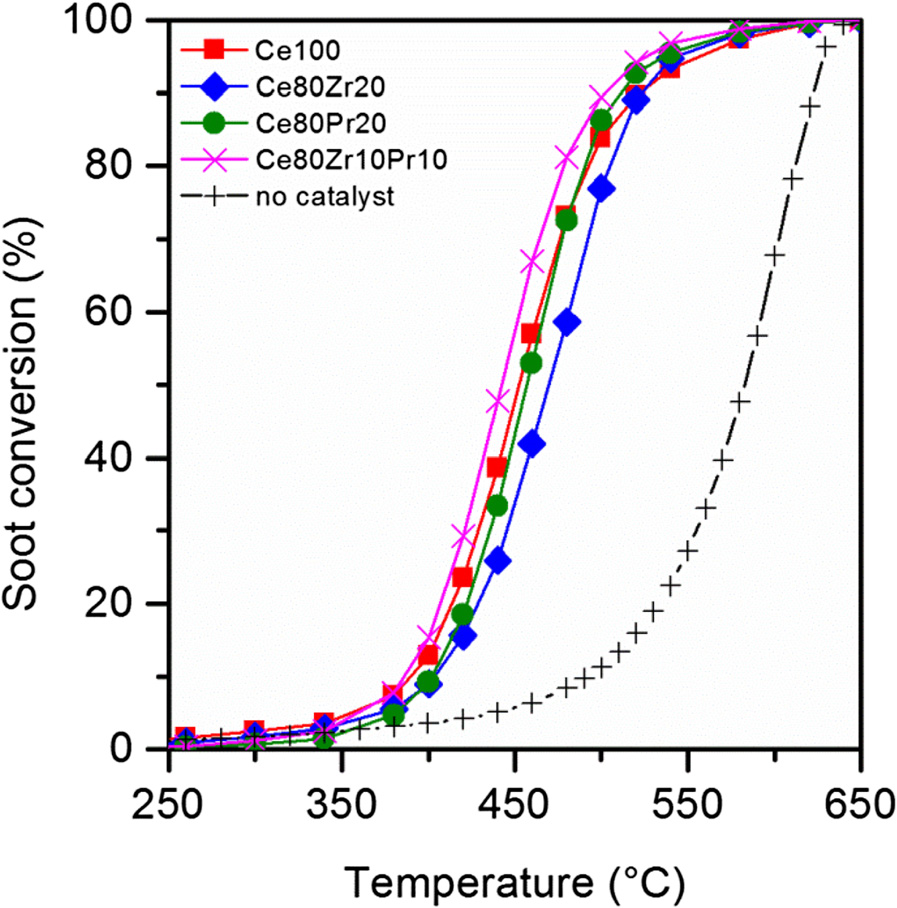
Soot conversion to CO_x_ profiles as a function of temperature for the prepared catalysts, under “tight” contact conditions

On the other hand, all the prepared oxide catalysts performed similarly with soot oxidation (Fig. 8). The temperatures at which half of carbon soot converted for Ce100, Ce80Zr20, Ce80Pr20, and Ce80Zr10Pr10 are 451, 469, 457, and 444 °C, respectively. Full data of the conversion temperatures are summarized in Table 8. The catalytic activity trend does not conform to the one for CO oxidation, although the tri-metallic oxide sample is still the most performing catalyst in the series. It seems that adding single dopants like Zr and Pr species does not promote enhanced catalytic activities. This is in fair agreement with previous measurements of Zr [15] and Pr [11] doping of SCS ceria, with respect to pure SCS ceria, although not verified in their co-presence. In contrast to this result, our previous works have also demonstrated that zirconium as well as praseodymium contributes positively to soot oxidation reaction over well-defined nanostructured catalysts (e.g., nanocubes and nanorods). This reconfirms our previous implication that soot oxidation reaction is highly structure-dependent [7].

**Table 8:** Results from soot oxidation over the prepared catalysts

Sample	*T*_10%_ (°C)	*T*_50%_ (°C)	*T*_90%_ (°C)
No catalyst	492	582	622
Ce100	393	451	521
Ce80Zr20	402	469	523
Ce80Pr20	401	457	512
Ce80Zr10Pr10	388	444	503

As previously seen through FESEM (see Fig. 2), our samples prepared via solution combustion technique possess similar structures irrespective of their metallic dopants. The primary particles constituting the overall structure are somewhat polycrystalline and lacking in low index planes such as (100) and (110) [4, 11, 33]. The presence of more uncoordinated atoms on these planes enhances the reactivity due to the instability of the atoms. Unlike CO, carbon soot is rather a passive reactant: it lacks mobility. Hence, the reaction strictly necessitates high surface reactivity of the catalyst as well as good catalyst-soot contact. This eventually justifies the aim of adjusting proper morphology of catalysts for soot oxidation [34].

**Fig. 9:**
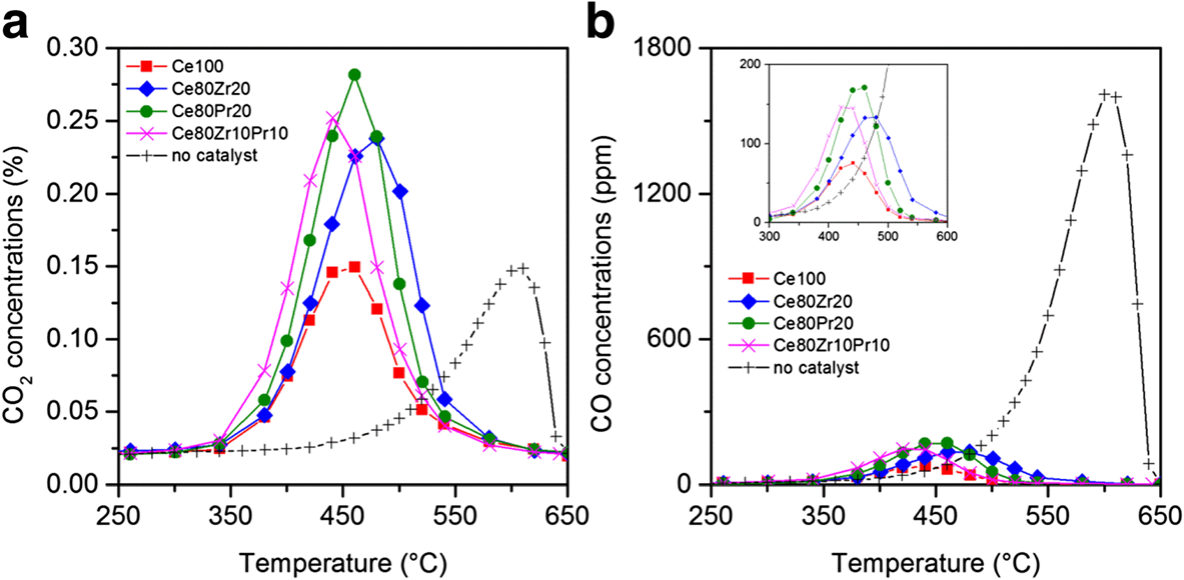
Profiles of CO_2_ (**a**) and CO (**b**) concentrations as a function of temperature for the prepared catalysts, under “tight” contact conditions

Finally, Fig. 9 shows the profiles of CO_x_ concentrations. The presence of catalysts increases the reaction selectivity to CO_2_, as the intensity of CO peak falls off at below 200 ppm. The introduction of dopants modifies the reaction selectivity to CO_2_ to a little extent; hence, the one for pure ceria sample (Ce100) is 94.7 %, while the one for the tri-metallic oxide sample (Ce80Pr10Zr10) is about 93.7 %.

## Conclusions

In this work, we present our tri-metallic oxide sample (Ce80Zr10Pr10) which has exhibited the highest catalytic activity of CO and soot oxidations among other oxides. Zr and Pr as dopants for ceria gives a significant impact on the catalytic activity of CO oxidation, as they enhance catalyst reducibility, redox behavior, and tune up catalyst physical properties (e.g., surface area and primary particle size). On the other hand, the effect of dopants is hardly observable in soot oxidation due to the morphological drawback resulted from solution combustion synthesis.
